# Towards the complete proteinaceous regulome of *Acinetobacter baumannii*

**DOI:** 10.1099/mgen.0.000107

**Published:** 2017-03-23

**Authors:** Leila G Casella, Andy Weiss, Ernesto Pérez-Rueda, J Antonio Ibarra, Lindsey N Shaw

**Affiliations:** ^1^​ Department of Cell Biology, Microbiology and Molecular Biology, University of South Florida, 4202 East Fowler Avenue, ISA 2015, Tampa, FL 33620-5150, USA; ^2^​ Instituto de Investigaciones en Matemáticas Aplicadas y en Sistemas, UNAM, Mérida, Yucatán, Mexico; ^3^​ Instituto de Biotecnología, UNAM, Cuernavaca, Morelos, Mexico; ^4^​ Laboratorio de Genética Microbiana, Departamento de Microbiología, Escuela Nacional de Ciencias Biológicas, Instituto Politécnico Nacional, Prolongación de Carpio y Plan de Ayala S/N, Colonia Santo Tomás, Delegación Miguel Hidalgo, CP, 11340 Mexico, DF, Mexico

**Keywords:** transcription factors, two-component systems, sigma factors, genome comparison, *Acinetobacter baumannii* AB5075

## Abstract

The emergence of *Acinetobacter baumannii* strains, with broad multidrug-resistance phenotypes and novel virulence factors unique to hypervirulent strains, presents a major threat to human health worldwide. Although a number of studies have described virulence-affecting entities for this organism, very few have identified regulatory elements controlling their expression. Previously, our group has documented the global identification and curation of regulatory RNAs in *A. baumannii*. As such, in the present study, we detail an extension of this work, the performance of an extensive bioinformatic analysis to identify regulatory proteins in the recently annotated genome of the highly virulent AB5075 strain. In so doing, 243 transcription factors, 14 two-component systems (TCSs), 2 orphan response regulators, 1 hybrid TCS and 5 σ factors were found. A comparison of these elements between AB5075 and other clinical isolates, as well as a laboratory strain, led to the identification of several conserved regulatory elements, whilst at the same time uncovering regulators unique to hypervirulent strains. Lastly, by comparing regulatory elements compiled in this study to genes shown to be essential for AB5075 infection, we were able to highlight elements with a specific importance for pathogenic behaviour. Collectively, our work offers a unique insight into the regulatory network of *A. baumannii* strains, and provides insight into the evolution of hypervirulent lineages.

## Abbreviations

HMM, hidden Markov model; MDR, multidrug resistant; NCBI, National Center for Biotechnology Information; RR, response regulator; TCS, two-component system; TF, transcription factor.

## Data Summary

The updated GenBank files for AB5075 and ATCC 17978, containing the revised transcription factor descriptions and annotations, have been deposited in Figshare: https://figshare.com/s/690a28e453bbe85eb683. Overviews and comparisons of TFs and two-component systems in various *Acinetobacter baumannii* genomes are shown in Tables S1 and S2 (available in the online Supplementary Material).

## Impact Statement

In the last two decades, the rise of *Acinetobacter baumannii* infections has presented an immense burden for patients and the public-health sector. Despite the increased number of reports describing clinical *A. baumannii* isolates expressing a wealth of virulence factors, and displaying resistance to most commonly used antimicrobials, little is known about the regulatory networks governing cellular behaviour. Indeed, very few regulatory elements, including transcription factors and two-component systems, have been described in *A. baumannii*. In this work, we identified all regulatory elements in the genome of the highly pathogenic AB5075 strain, and assessed their conservation across several clinical isolates and a laboratory isolate. Given the importance of regulatory elements, and their potential as therapeutic targets, this comprehensive analysis provides unique insight into conserved, yet uncharacterized, regulators, and those present only in pathogenic strains. These findings represent a foundation for further investigation towards the importance of these novel regulatory elements, and their contribution to *A. baumannii* pathogenicity.

## Introduction

*Acinetobacter baumannii* is a Gram-negative pathogen that is becoming increasingly problematic due to its ability to survive on fomite surfaces, resist the action of common disinfectants and evade treatment with antimicrobial agents [1, 2]. Consequently, this organism causes severe infections in both hospital and community settings, including pneumonia, skin and soft-tissue infections, urinary-tract infections, endocarditis, and meningitis [1, 3–6].

Most studies exploring the mechanisms of disease causation by this important pathogen have been performed using two *A. baumannii* strains, ATCC 19606 and ATCC 17978, both isolated in the 1950s. However, several genomic differences between these strains and more recent clinical isolates make both suboptimal for the study of pathogenesis. For example, each of these strains lacks the 86 kb pathogenicity island AbaR1 that harbours genes important for resistance to metal ions and an array of antibiotics [7]. This renders both strains susceptible to common therapeutics that are ineffective against the antibiotic-resistant and highly virulent strains currently found in most hospital settings [8–10]. Furthermore, recent clinical isolates display extensive genomic variation, as well as hypervirulent phenotypes, when compared to their historic counterparts, highlighting the rapid evolution of contemporary isolates [11].

In line with this, a recent study sought to characterize modern clinical strains of *A. baumannii* using genetic approaches, alongside a murine model of pneumonia and a *Galleria mellonella* model of infection [12]. This study identified strain AB5075, a multidrug-resistant (MDR) wound isolate recovered from a patient at the Walter Reed Army Medical Center, USA [13], as being highly virulent, suggesting the potential for novel genes and regulatory mechanisms that enable this strain to colonize, disseminate and persist in different infection sites [14].

Amongst the genes found to be important for pathogenicity in *A. baumannii*, several transcriptional regulators have been identified. This is perhaps unsurprising, as the production of virulence determinants in *A. baumannii*, much like in other bacteria, is a finely tuned process that allows for adaptation to changing environmental conditions and survival in specific niches. This tight regulation of gene expression is, amongst others things, controlled by transcription factors (TFs, also known as one-component systems), which are classified into several families, commonly generally based on two key features: (i) a DNA-binding domain that interacts with enhancer, silencer or promoter regions; and (ii) a trans-acting domain that often serves as a sensor, receiving signals such as protein–protein interaction or the binding of small molecules [15]. Similarly to TFs, two-component systems (TCSs), which typically consist of a membrane-embedded sensor kinase and a cytoplasmically located response regulator (RR), can react to environmental stimuli and trigger a cellular response by influencing the transcriptional process [16]. Finally, alternative σ factors can also influence regulatory networks by facilitating DNA-dependent RNA polymerase (RNAP) recognition of unique promoters [17]. Each of these different regulatory elements (TFs, TCSs, σ factors) interacts with RNAP in specific and discrete ways, modulating promoter recognition and transcriptional initiation of target genes [15].

To date, only a handful of TFs have been characterized in *A. baumannii*, including AdeL, a LysR-type regulator, and AdeN, a TetR-like regulator, controlling expression of the AdeFGH and AdeIJK efflux pumps, respectively [18–20]. Additionally, the ferric-uptake regulator (Fur) has been described as controlling expression of genes involved in siderophore production [21, 22]. A fourth TF, SoxR, is a MerR-like regulator governing transcription of the AbuO outer-membrane protein, which is important for resistance to osmotic and oxidative stress [23]. Finally, Zur is a Fur-like regulator identified as being critical for zinc homeostasis in a mouse model of *A. baumannii* infection [24]. With respect to TCSs, only five have thus far been characterized in *A. baumannii.* The first, BfmSR, controls production of capsular exopolysaccharides as well as pilus assembly, and consequently, cell attachment and biofilm formation [25, 26]. Additionally, the PmrAB TCS has been described as sensing low Mg^2+^ concentrations, as well as cationic antibiotics such as polymyxin B [27]. Another two TCSs, AdeRS and BaeRS, were shown to be connected with antibiotic exposure, both controlling the expression of AdeABC, a major efflux pump conferring resistance to tigecycline [28–30]. Finally, GacS, a hybrid TCS, which interacts with GacA, an orphan RR, controls the phenylacetic acid catabolic pathway, as well as genes involved in biofilm formation, pilus synthesis and motility [31]. To date, there are no studies describing the role of alternative σ factors in *A. baumannii*.

Previously, we have documented the global identification and curation of regulatory RNAs in *A. baumannii* strain AB5075 [32]. In the context of proteinaceous regulators, however, their essential role in cellular physiology and pathogenesis is still largely unexplored. As such, our goal was to perform a comprehensive evaluation of the AB5075 genome to identify and classify every TF, TCS and σ factor. Following this, we compared the distribution of these elements among susceptible and MDR strains. This comparison provides a unique insight into *A. baumannii*-specific regulators, and sheds light onto the influence of TFs in the evolution of pathogenesis in this organism.

## Methods

### Identification of TFs

To identify TFs in *A. baumannii* AB5075, we used a combination of information sources and bioinformatics tools. The complete set of TFs identified in *Escherichia coli* [33], *Bacillus subtilis* [34] and *Staphylococcus aureus* [35] were used as seeds to search for homologues in the complete genome of *A. baumannii* strains using blastp searches (with an *E* value ≤10^−3^ and a coverage of ≥60 %). In addition, we used a battery of hidden Markov model (HMM) profiles associated with the three bacterial reference datasets to identify potential TFs not resolved by blastp searches. Finally, pfam, Superfamily and CD-search were used to assign evolutionary families, and to exclude proteins with no regulatory activity (i.e. false positives). In all cases, an *E* value ≤10^−3^ was used as a cut-off. For each TF identified in *A. baumannii* AB5075, a blastn and blastp search against other *A. baumannii* genomes was performed. For a protein to be considered a homologue, a BLASTP *E* value ≤10^−20^ and coverage of ≥60 % relative to the seed sequence was required. Further classification of TF families was performed using pfam database annotations and the database of Clusters of Orthologous Groups of proteins (cogs); all of which were verified using blast searches against annotated protein families. Assignments of putative function to uncharacterized regulatory elements in AB5075 were performed using a combination of blastp and literature searches of hits corresponding to well-characterized proteins. An *E* value ≤10^−10^ and coverage ≥30 % of aligned proteins was used as a cut-off for this analysis. Updated genome annotations files for AB5075 and ATCC 17978, containing the results of our bioinformatics analyses, have been deposited in Figshare (https://figshare.com/s/690a28e453bbe85eb683).

### Proteomes analysed

In order to determine conservation of TFs in the genome of AB5075, we analysed six *Acinetobacter* genomes. These were: *A. baumannii* AB5075 (https://dx.doi.org/10.6084/m9.figshare.1592959.v1); *A. baumannii* AB0057 (GenBank accession no. CP001182); *A. baumannii* AB307 0294 (GenBank accession no. NC_011595); *A. baumannii* ACICU (GenBank accession no. NC_010611); *A. baumannii* ATCC 17978 (GenBank accession no. CP000521.1); and *A. baumannii* AYE (GenBank accession no. NC_010410). Unless noted otherwise, sequences were downloaded from the National Center for Biotechnology Information (NCBI) ftp server (www.ncbi.nlm.nih.gov/genome).

### Identification of σ factors

In order to identify σ factors present in AB5075, we used the genome sequences of *E. coli* K-12 MG1655 (GenBank accession no. NC_000913) and *Pseudomonas aeruginosa* PAO1 (GenBank accession no. NC_002516.2). Sequences were downloaded from the NCBI ftp server (www.ncbi.nlm.nih.gov/genome). A search for homologues in the genome of AB5075, and subsequent conservation analysis in the six other *A. baumannii* strains, was performed using blastp searches (with an *E* value ≤10^−20^ and coverage of ≥60 %). Further alignments were generated using CLC Genomics Workbench (version 7.6.1; CLC bio).

## Results and Discussion

### Identification of TFs in the *A. baumannii* 5075 genome

**Fig. 1.**
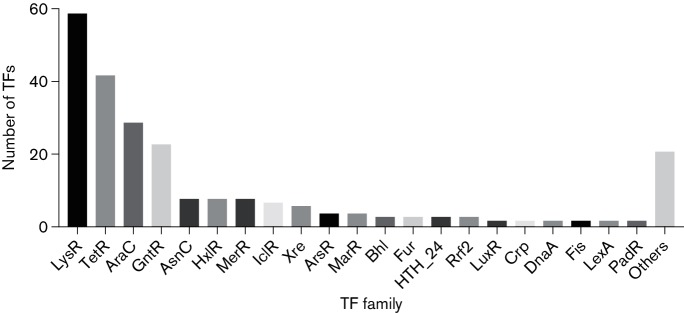
Classification of TFs identified in *A. baumannii* AB5075. The grouping of TFs into families was performed by blastp analysis. Groups that contained only one protein were combined under ‘others’.

In order to enhance our understanding of regulatory networks within *A. baumannii*, we performed a genome-wide analysis of TFs present in strain AB5075. This strain was chosen because it has a fully annotated genome, is hypervirulent in animal models of infection, is resistant to many commonly used antibiotics, and belongs to one of the three main clonal lineages most prevalent in hospital outbreaks worldwide [14, 36–38]. Accordingly, we surveyed the entire genome of AB5075 using HMM profiling to predict the presence of TFs. These findings were manually curated by validation of protein sequences against pfam libraries and by blast searches against the NCBI database. This resulted in the identification of 243 TFs (Table S1, available in the online Supplementary Material). These TFs were classified into 42 different families (Fig. 1, Table S1), with the majority corresponding to the LysR (59) and TetR families (42). One of the regulatory proteins identified was DnaA (ABUW_0001). In *E. coli*, DnaA has a dual role, functioning both as an initiator of replication and also acting as a transcriptional regulator [39, 40]. This latter function includes the repression and activation of several genes, including *guaA, dam, rpoH, ftsA and mioC*, which are involved in metabolic functions, chromosomal replication, cell division and stress response [39]. Therefore, the corresponding DnaA protein of *A. baumannii* was included as a TF in our study.

Notably, and as suggested above, of all the TFs identified in AB5075, only a limited number have been characterized thus far in any *A. baumannii* isolate (Table S1). These include: (i) the TetR and LysR proteins AdeN (ABUW_1731) and AdeL (ABUW_1338) [19]; (ii) two members of the Fur family, Fur (ABUW_3033) and Zur (ABUW_3741) [21, 22, 24]; and (iii) the MerR-like protein SoxR (ABUW_2555) [23].

As functions for the majority of regulators identified in our screen have not been reported in *A. baumannii*, we performed a blastp analysis and literature search for similar proteins to query sequences, thereby allowing us to propose theoretical functions (Table 1). In so doing, we were able to assign putative functions to 29 TFs, corresponding to AraC, ArsR, Bhl, Crp, DnaA, Fis, GntR, IclR, LysR, LytR, MarR, MerR, NrdR, Rrf2 and TetR family proteins, based on characterized factors with strong sequence similarity in the NCBI database. This included TFs that function in: the response to iron starvation, the production of exotoxins and utilization of aromatic compounds as carbon sources, as well as more general metabolic processes (Table 1). Notably, despite the large amount of LysR and TetR proteins identified, very few were found to have homologues in other organisms. One such element, ABUW_2970 (TetR family), displayed a sequence identity of 81 % to the TF BetI, which acts as a repressor of two choline transporters, and the *betIBA* operon, which regulates the choline oxidation pathway, in *Acinetobacter baylyi* (Table 1) [41].

**Table 1.** Putative functions of TFs in *A. baumannii* AB5075 Shaded rows indicate genes required for *A. baumannii* infection of *G. mellonella* [12]. T3SS, Type III secretion system.

Protein ID	Family	Comment	Identity (*E* value)	Reference
ABUW_3565	AraC	Regulator of methylation damage in *E. coli*	32 % (2*e*−49)	[93]
ABUW_2370	ArsR	Involved in arsenic detoxification in *E.coli*	52 % (3*e−*30)	[94]
ABUW_3668	ArsR	Regulator of the *ars* operon in *E. coli*	59 % (4*e−*35)	[95]
ABUW_2198	Bhl	Nucleoid organization and regulation in *E. coli*	71 % (8*e−*46)	[96]
ABUW_2241	Bhl	Required for site-specific recombination system expression in *E. coli*	56 % (6*e−*40)	[42]
ABUW_3279	Bhl	Integration host factor (IhfA) in *E. coli* controlling type 1-fimbrial expression *(fimA)*	68 % (5*e−*47)	[43]
ABUW_2741	Crp	Regulator of genes involved in the production of exotoxin and secretion systems (T3SS) in *P. aeruginosa*	55 % (9*e−*83)	[44, 97]
ABUW_0001	DnaA	Regulates initiation of bacterial replication in *E. coli*	48 % (2*e−*159)	[98]
ABUW_1533	Fis	Homeostatic regulator of DNA topology in *E. coli*	59 % (8*e−*33)	[45, 46]
ABUW_3813	GntR	Regulator of genes involved in transport and catabolism of l-lactate in *E. coli*	48 % (4*e−*78)	[99, 100]
ABUW_2775	GntR	Repressor for utilization of vanillate in *A. baylyi* ADP1	84 % (6*e−*141)	[101]
ABUW_0075	GntR	Regulator of histidine utilization genes in *Brucella abortus*	31 % (7*e−*42)	[102]
ABUW_1848	IclR	Controls protocatechuate degradation in *A. baylyi* ADP1	82 % (3*e−*172)	[47, 48]
ABUW_2488	IclR	Regulation of *pobA* in response to *B*-hydroxybenzoate in *A. baylyi* ADP1	82 % (2*e−*170)	[103]
ABUW_0067	IclR	Repressor of an aromatic catabolic pathway in *Pseudomonas putida*	31 % (7*e−*32)	[104]
ABUW_1599	LysR	Involved in regulation of genes responsible for swarming in *P. aeruginosa*.	47 % (1*e−*77)	[105]
ABUW_1878	LysR	Repressor of benzoate catabolism in *P. putida*	39 % (6*e−*65)	[106]
ABUW_2709	LysR	Regulation of benzoate degradation in *A. baylyi* ADP1	65 % (2*e−*149)	[107, 108]
ABUW_2849	LysR	Inhibitor of DNA replication in *E. coli*	36 % (3*e−*34)	[109]
ABUW_3471	LysR	Regulation of 3-phenylpropionic acid catabolism in *E. coli*	43 % (1*e−*75)	[110]
ABUW_1016	LysR	Positive regulation of sulfate starvation inducible genes	52 % (2*e−*12)	[111–113]
ABUW_3615	LytTR	Regulator of alginate biosynthesis in *P. aeruginosa*	47 % (2*e−*78)	[53]
ABUW_3790	MarR	Leucine-responsive regulatory protein (Lrp) in *E. coli*	57 % (1*e−*59)	[114]
ABUW_2706	MerR	Regulator of copper export in *E. coli*	41 % (2*e−*42)	[115]
ABUW_3015	MerR	Positive regulation of *slpA*, resulting in excision of cryptic prophage (CP4–57) in *E. coli*	40 % (8*e−*04)	[116, 61]
ABUW_3665	MerR	Cadmium-induced regulator in *P. aeruginosa*	49 % (3*e−*36)	[117, 118]
ABUW_3653	NrdR	Regulator of ribonucleotide reductases operons in *E. coli.*	55 % (3*e−*64)	[54]
ABUW_2201	Rrf2	Regulator of iron–sulfur clusters in *E. coli*	54 % (2*e−*49)	[49]
ABUW_2970	TetR	Choline-responsive repressor in *A. baylyi*	81 % (9*e−*130)	[41]

**Fig. 2.**
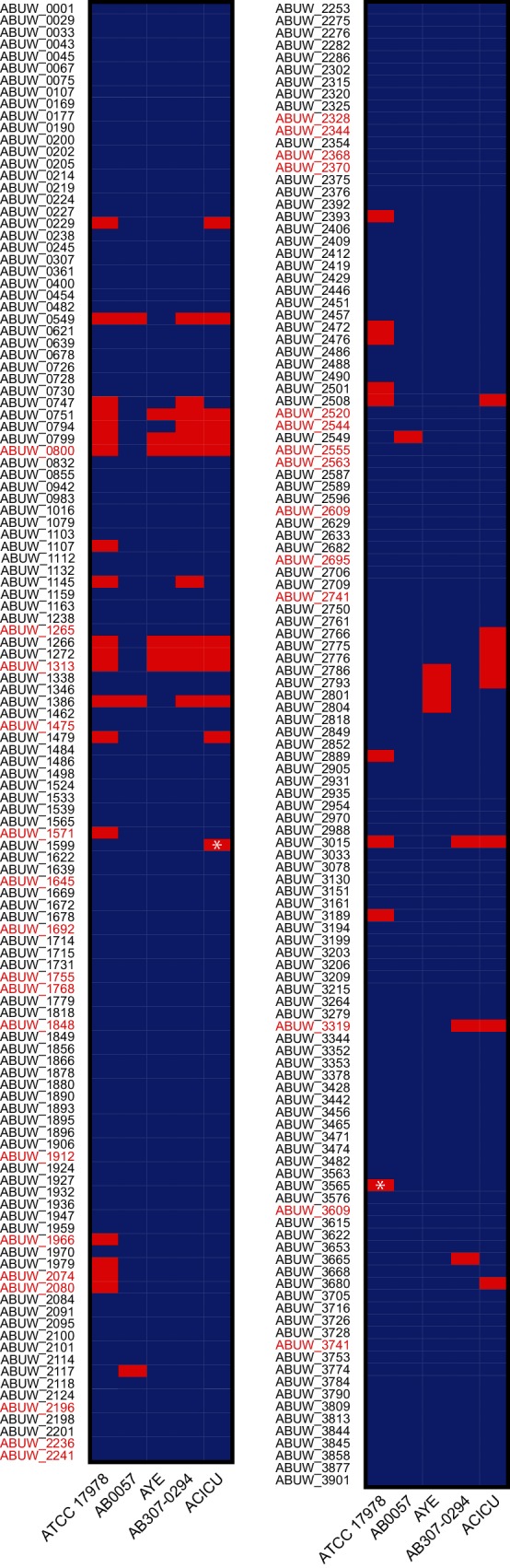
Conservation of TFs across *A. baumannii* strains. Homologues to AB5075 regulatory proteins were identified as outlined in Methods. A blue box denotes the presence of an homologue, while red boxes mark the absence of a given regulator. An asterisk highlights frameshift mutation(s) in the homologous ORF. Gene names that are in red font highlight factors that have been shown to be essential for AB5075 infection in a worm model [12].

In an attempt to provide some pathogenic context to this curation of TFs, we compared our findings to recent work by Gebhardt and colleagues, who screened a transposon insertion sequence library (TnSeq) of AB5075 in a *G. mellonella* infection model [12]. This resulted in 31 TFs reported as being essential for growth in their worm model (Table S1, highlighted in Fig. 2). Interestingly, among the TFs with putative functions listed in Table 1, four were reported to be essential for survival of AB5075 in *G. mellonella* (shaded in grey in Table 1). This group includes ABUW_2241, a member of the Bhl family required for regulation of genes controlling several cellular processes, including lambda site-specific recombination, in *E. coli* [42, 43]. Additionally, a member of the Crp family (ABUW_2741) was found to be important for infection in the worm model. Of interest, a homologue to this protein serves to regulate the type III secretion system in *P. aeruginosa* [44]. Finally, two proteins, ABUW_1848 and ABUW_2370, members of the IclR and ArsR families of transcriptional regulators, respectively, were also required for *A. baumannii* virulence. A homologue of ABUW_2370 has been reported to control expression of genes involved in regulating arsenic detoxification in *E. coli,* whilst an ABUW_1848 homologue controls aromatic catabolism in *A. baylyi* ADP1. [45–49].

### TF distribution in AB5075

#### Core regulatory elements

Next, we sought to investigate the conservation of AB5075 TFs in other *A. baumannii* strains. We hypothesized that the absence of certain TFs in less pathogenic strains, when compared to their hypervirulent counterparts, may aid in explaining their decreased ability to infect mammalian hosts. As such, we analysed the laboratory strain ATCC 17978, which is susceptible to the majority of antibiotics used for *A. baumannii* infections, as well as four clinical isolates, including the drug susceptible AB307-0294 strain, and three MDR isolates: AB0057, AYE and ACICU [10, 50–52]. In so doing, we found a set of 202 TFs to be conserved in each genome; thus, presenting the core regulatory elements of these species (Fig. 2, Table S1). Within this set, only 22 TFs had putatively assigned functions. For these TFs, homologues in other organisms are involved in the regulation of metabolism, detoxification and virulence, suggesting that they may fine tune the expression of essential genes important for basal and conserved physiological processes [49, 53–60].

Next, we investigated the number of conserved TFs in relation to the genome size for each *A. baumannii* strain. Despite the fact that ATCC 17978 and AB5075 both have a genome size of approximately 4.0 Mbp, the conservation of TFs between these two strains is only 88.5 %, reflecting significant potential genomic alterations due to horizontal gene transfer, insertions or deletions. Conversely, AB0057, AYE, AB307-0294 and ACICU showed TF conservation of 98.4, 95.9, 94.2 and 91.4 %, respectively (Table 2), indicating a closer evolutionary proximity to AB5075. An important note is that, for these conservation analyses, we used an AB5075 centric view, in that we did not assess TFs present in other strains but not in AB5075 itself. As such, there remains the possibility that the loss of certain elements, which might be common to strains other than AB5075, could help to explain the hypervirulence of the latter. Nevertheless, as we were primarily interested in understanding the physiology of AB5075, we placed an emphasis on the specific regulatory elements in this strain.

**Table 2.** Comparison of genome size and the presence of homologues to AB5075 regulators in various *A. baumannii* strains

Strain	Genome size (nt)	No. of conserved TFs	Conservation relative to AB5075 (%)
AB0057	4 050 513	239	98.4
AYE	3 936 291	233	95.9
AB307-0294	3 760 981	229	94.2
ACICU	3 904 116	222	91.4
ATCC 17978	3 857 743	215	88.5

#### Non-conserved elements

In contrast to the TFs conserved across each of the different isolates, we found several proteins to be absent in one or more of the investigated strains (Fig. 2, Table S1). For example, ABUW_3015, a putative member of the MerR family of regulators, is only conserved in AB0057 and AB307-0294. Of note, ABUW_3015 showed 40 % sequence identity to a TF in *E. coli*, AlpA, previously characterized as a positive regulator of *slpA*, a gene that is part of a cryptic prophage suggested to be involved in biofilm formation (Table 1) [61]. Importantly, the presence of phage-like regions in the chromosome of several *A. baumannii* clinical strains has been previously reported, including for AB0057 and AB5075; however, their significance has yet to be elucidated [37, 50]. Likewise, ABUW_1599, a LysR-type regulator, was absent in the ACICU strain. According to the putative function assigned in this work (Table 1), ABUW_1599 may be involved in the regulation of swarming motility. Interestingly, this phenotype has been reported to be absent in ACICU, although the genes encoding a type IV pilus are present in ACICU [62].

#### Non-conserved elements that are important for virulence

The recent *in vivo* screening of an AB5075 transposon library using a *G. mellonella* infection model [12] identified several TFs that were found not to be conserved across *A. baumannii* strains (Table S1) [12, 63]. Among these are ABUW_2074, a member of the Fur family, which is absent in the non-pathogenic strain ATCC 17978. Interestingly, this strain also lacks a heme uptake system and heme oxygenase, which have both been shown as necessary for virulence [64]. Given that Fur family proteins are known to be involved in the regulation of iron uptake, and that ABUW_2074 is conserved in all four clinical *A. baumannii* strains studied herein, this may suggest a central role for ABUW_2074 in controlling nutrient acquisition in the host [64, 65]. Additionally, ABUW_1966, a member of the LysR family, is absent in ATCC 17978. Interestingly, ABUW_1966 has been shown to be necessary for resistance to antibiotics targeting cell-wall synthesis, and is required for growth in *G. mellonella* [12]. In this context, ATCC 17978 is a drug-sensitive strain, possibly suggesting ABUW_1966 may have a role in regulating genes required for antimicrobial resistance.

### TCSs in AB5075

In addition to TFs, another layer of regulation found in bacteria is mediated by TCSs. These systems typically combine a sensor kinase protein that receives a signal following a specific stimulus, and transduces this to a RR protein via phosphorylation, resulting in altered gene expression [66]. Given that a TF might interact with a sensory protein and serve as a RR, we combined HMM profiling with blastp analysis using a list of TCSs corresponding to the *P. aeruginosa* PAO1 genome to detect these elements in AB5075. We identified 14 RR proteins encoded together with a sensor kinase protein. This set of 14 TCSs includes: (i) 11 members of the OmpR family; (ii) 2 members of the HTH_8 family; and (iii) 1 member of the LuxR family (Table S2). This latter TCS corresponds to a sensor protein (ABUW_2427) that possesses both histidine kinase and RR domains, and is encoded adjacent to the RR protein ABUW_2426. Given that the hybrid sensor kinase ABUW_2427 and RR ABUW_2426 are localized within the same operon it has been included in the set of 14 TCSs. Of note, from the 14 RR/sensor pairs identified, only 4 have previously been studied in *A. baumannii* (Table S2). ABUW_0608/ABUW_0609 and ABUW1972/ABUW1973 have previously been named BaeSR and AdeRS, respectively, and described as having regulatory roles in the expression of efflux pumps [30, 67]. Likewise, ABUW_0828/ABUW_0829 corresponds to the PmrAB TCS that was found to confer resistance to the cationic antimicrobial colistin [27]. Finally, ABUW_3180/ABUW_3181 was characterized as TCS BfmSR, which controls biofilm formation, motility and exopolysaccharide production, and is required for pathogenicity in a *G. mellonella* infection model [12, 25–27, 30, 67].

As with our analysis of TFs, we next searched for homologues to the remaining 10 TCSs to identify those that may have been characterized in other species. In so doing, we assigned putative functions to seven additional elements (Table 3). Of these, two members of the HTH_8 family, ABUW_1732 and ABUW_3641, showed sequence similarity to hitherto characterized proteins. ABUW_1732 shows 64 % identity to NtcR, a RR that controls the *nifLA* operon, which is required for nitrogen assimilation in *Klebsiella pneumoniae*; while ABUW_3641 displays 51 % identity to a type IV fimbriae RR in *P. aeruginosa* [55, 68]. The five remaining factors were all OmpR RRs, and had similarity to systems controlling the response to phosphate, heavy metals, osmotic stress or the expression of flagella (Table 3). Of note, although not yet characterized, ABUW_1514/1515 was found to be required for growth in *G. mellonella*, which implies its importance for AB5075 pathogenicity (Table S2) [12].

**Table 3.** List of TCSs found in AB5075 with putatively assigned functions

Protein ID	Family	Comment	Identity (*E* value)	Reference
ABUW_1732	HTH_8	Regulator of nitrogenase synthesis in *K. pneumoniae*	64 % (0)	[55, 119]
ABUW_3641	HTH_8	Regulator of type IV fimbriae in *P. aeruginosa*	51 % (2*e−*165)	[68]
ABUW_0106	OmpR	Regulator of the PhoB regulon during phosphate starvation in *E.coli*	62 % (6*e−*104)	[120]
ABUW_0257	OmpR	Two-component OmpR–EnvZ regulator that senses osmotic stress in *E. coli*	69 % (2*e−*117)	[121], [122]
ABUW_1506	OmpR	Regulator of genes involved in resistance to cadmium and zinc in *Burkholderia pseudomallei*	53 % (6*e−*89)	[71]
ABUW_1585	OmpR	Involved in K^+^ ion transport regulation in *E. coli*	42 % (4*e−*67)	[123]
ABUW_3323	OmpR	RR involved in copper resistance in *E. coli*	62 % (2*e−*109)	[124, 125]

In addition to TCSs, we also identified two orphan RRs that were not associated with a histidine kinase, ABUW_0180 and ABUW_3639, which are members of the LuxR and GerE families, respectively. Interestingly, ABUW_3639 has been previously characterized as GacA, an orphan RR of the phenylacetic acid catabolic pathway, which interacts with a hybrid sensor kinase GacS [31]. In concert both elements control expression of the *csu* operon, which is involved in pilus synthesis, required for virulence in *A. baumannii* ATCC 17978, as well as genes involved in biofilm formation. [31]. Lastly, ABUW_3306 was identified as a hybrid TCS, harbouring sensory and regulatory domains. Upon analysis, it was determined that the protein showed 100 % identity to GacS in ATCC 17978 [31].

### TCS distribution in AB5075

Of the 14 TCS RRs identified in this study, only 1 was found not to be conserved in all the five strains used in this analysis. This was ABUW_ 3641, a member of the HTH_8 family, which was marked as absent (Table S2) in the laboratory strain ATCC 17978 based on a combination of protein and nucleotide blast analysis (discussed further below). The conservation of ABUW_3641 may reflect its role in regulating genes required for twitching motility, which is a common phenotype reported in *A. baumannii* strains, with the exception of ATCC 17978 [62, 69, 70]. In contrast, the remaining 13 TCS RRs, 2 orphan RRs and the hybrid TCS identified were common to all *A. baumannii* strains. Of note, seven of the conserved TCS RRs had predicted functions, including the response to osmotic stress, heavy metal efflux and the regulation of motility [71].

### σ factors in AB5075

Along with TFs, σ factors provide another, and perhaps more basal, level of regulation, guiding RNAP to unique promoters to initiate transcription of specific genes in response to external stimuli [17]. Given their vital regulatory role, we next explored the genome of AB5075 to identify potential σ factors genes. To do this, we generated a list of known σ factors present in the genomes of the *P. aeruginosa* strain PAO1 and *E. coli* strain K-12, both of which contain multiple σ factors that have been well described [72–79]. The amino acid sequence for each of these elements was then used in blastp searches against the AB5075 genome. Resulting hits were subsequently analysed using the pfam database to determine conservation of domains inherent to σ factor activity. Alignments of each putative AB5075 σ factor were then generated for *E. coli* K-12 and *P. aeruginosa* PAO1 homologues to identify specific active site residues required for σ factor function.

**Fig. 3.**
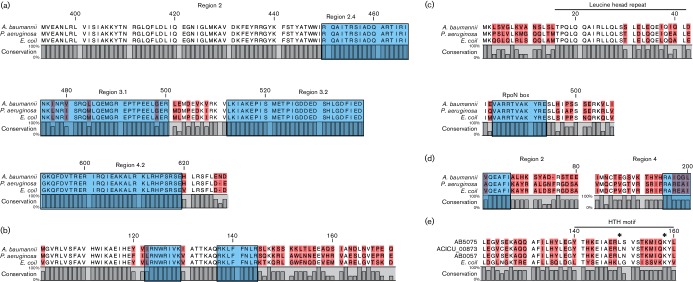
Alignment of σ factors from *E. coli*, *P. aeruginosa* and *A. baumannii.* Multiple alignments were performed for σ factors found in *A. baumannii* AB5075, *E. coli* and *P. aeruginosa*. (a) Conservation in regions 2.4 and 4.2, which are essential for promoter recognition, as well as region 3 (all indicated by blue boxes), were observed. (b) Two conserved amino acid sequences critical for binding to DnaK (blue boxes) were noted for all three RpoH proteins. (c) Several leucine residues, within a conserved heptad motif, and a conserved amino acid sequence only found in σ54 family proteins (blue box), were identified in RpoN homologues. (d) Blue boxes indicate a conserved amino acid sequence within regions 2 and 4 of RpoE, important for transcription of *rpoE* and promoter recognition in general. (e) Asterisks indicate conservation of a leucine residue essential for FecI–FecR interaction, and a lysine residue critical for binding to the β subunit of RNAP. Throughout this figure, pink boxes represent amino acids that display divergence between the compared sequences.

Consequently, we identified five putative σ factors in AB5075, including ABUW_0862, which is the *A. baumannii* RpoD (σ^A^) homologue. Specifically, ABUW_0862 showed sequence identity of 62 and 63 % to RpoD of *E. coli* and *P. aeruginosa*, respectively. As shown in Fig. 3(a), alignment of amino acid sequences revealed the conservation of regions 2.4 and 4.2, which is in agreement with the function of these domains in recognizing the −10 and −35 sequences of housekeeping genes. The inhibitory domain, region 1, and the RNAP binding domain, region 3, were also present [80]. Other than σ^A^, we identified ABUW_1375 as having 64 % identity to RpoH of *P. aeruginosa* and 59 % to RpoH from *E. coli.* Comparative alignments for RpoH proteins revealed the conservation of two motifs (QRKLFFNLR and LRNWRIVK) located in region 2.4, both reported binding sites for DnaK, a protein chaperone that controls RpoH stability (Fig. 3b) [81]. A third putative σ factor, ABUW_3253, was found to have conserved regions characteristic of RpoN, a member of the σ^54^ family, involved in regulating nitrogen metabolism and bacterial virulence [76]. Two distinct regions in ABUW_3253 were found to be similar to the RpoN protein of *E. coli* (Fig. 3c): region 1 of ABUW_3253 contains several leucine residues within a heptad motif previously reported to be important for recognition of −12 promoter elements; whilst a signature σ^54^ amino acid sequence (ARRTVAKYRE), also known as the RpoN box, required for DNA binding, was also found [76, 82, 83]. Lastly, ABUW_0988 and ABUW_2987 were found to have conserved regions in domains σ_2_ and σ_4_ found in the extracytoplasmic σ factors RpoE and FecI, which have been reported to function in cell envelope stress and iron acquisition, respectively [84, 85]. Alignments of ABUW_0988 with RpoE of *E. coli* and AlgU from *P. aeruginosa*, a homologue of RpoE (Fig. 3d), showed conservation of the VQEAFI sequence in region 2, which is thought to be critical for transcription of *rpoE*. Further to this, several amino acid residues, such as arginine, lysine, leucine and isoleucine, were found in region 4, which are believed to be important for recognition of −35 promoter regions and binding of anti-σ factor proteins [75, 86, 87]. With regards to ABUW_2987, it is noteworthy that it is located in an apparent operon that has similar organization to the *fecABCDE* operon required for ferric citrate transport in *E. coli* [88]. Interestingly, alignment of ABUW_2987 with FecI of *E. coli* identified two conserved residues (L146 and K155), both within a helix-turn-helix motif in region 4.2, that have been reported as being essential for FecI–FecR interaction and binding to the β′ subunit of RNAP, respectively (Fig. 3e) [84].

We next explored conservation of AB5075 σ factor proteins in the genomes of other *A. baumannii* strains. We found that four of the five σ factors were ubiquitously conserved, but, quite surprisingly, the FecI-like protein ABUW_2987 was absent in all *A. baumannii* strains with the exception of two MDR clinical isolates, AB0057 and ACICU [52]. Of note, FecI is found in AB5075 within a cluster of eight genes, encoding protein homologues of FecR, TonB and HemO, which encode a cytoplasmic protein sensor of ferric citrate, a TonB-dependent outer membrane transport protein and a heme oxygenase, respectively. Similarly, this cluster is also absent in ATCC 17978, AB307-0294 and AYE strains. Interestingly, this locus is conserved in ACICU and AB0057, as well as in *A. baumannii* LAC-4, a virulent isolate with tolerance to chelators of ferric iron [64]. It is possible that the lack of conservation of this cluster in some *A. baumannii* strains is due to recent acquisition via lateral transfer. This notion is supported by the idea that possession of this operon might represent a mechanism for acquisition of heme as an alternative iron source, providing an advantage for survival in iron-limited environments, such as during infection, for virulent clinical strains [89–91].

### Inconsistent annotations and genomic divergence in *A. baumannii* strains

Given that our study is largely reliant on accurate genome annotation files, and that we have previously reported inconsistencies in prokaryotic repositories of this type [32, 92], we took an additional approach to our analysis so as to minimize the influence of annotation inaccuracies on our investigations. As such, alongside protein/annotation-based searches, we also performed nucleotide blast analyses (Table S1). The rationale for this was to identify genomic regions with nucleotide conservation for a given TF-encoding ORF, regardless of whether automated systems had annotated a protein. While generally the quality of annotation files for the strains used was high, allowing a comprehensive inter-genome comparison, we did uncover a number of inconsistencies. Firstly, we identified several loci that showed extensive nucleotide conservation towards annotated AB5075 TF ORFs, but that did not have an annotation at the corresponding position. We also discovered regions that, despite retaining nucleotide conservation, had differing length gene annotations between the various strains, indicating the use of alternative start codons. Finally, we noted regions where mutations (e.g. frameshifts) had occurred, leading to the presence of novel stop codons, and thus partial annotations and/or altered protein sequences.

**Fig. 4.**
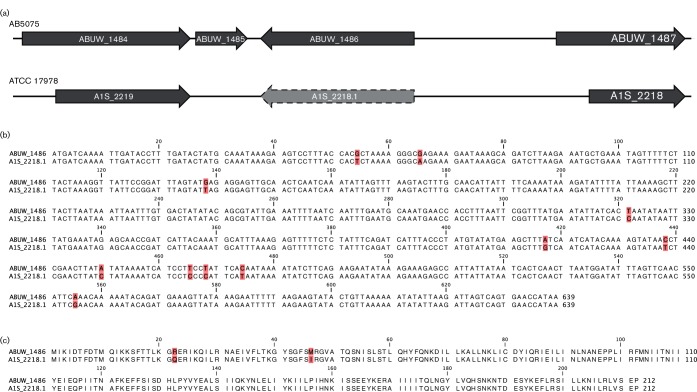
The influence of automated annotation systems on genomic content. (a) Genomic context of the AB5075 regulator ABUW_1486 and its homologous region in ATCC 17978. The current annotations for each genome are shown by dark grey arrows, whilst the light grey arrow with a dashed outline is a possible missing annotation (A1S_2218.1 in ATCC 17978). Although not annotated in the genome, this region shows high conservation at the (b) nucleotide and (c) amino acid level when compared to the ABUW_1486 protein and its coding region. Differences between the compared sequences are highlighted by pink boxes.

With respect to this first category, AB5075 ABUW_1486 is annotated as a TetR-type regulatory protein; however, no clear homologue is present in ATCC 17978. Using a nucleotide blast, we identified a large, intergenic and unannotated region of 1656 nt in ATCC 17978 that displayed major sequence homology to the AB5075 ABUW_1486 locus (Fig. 4a, b). Using bioinformatics analyses (http://web.expasy.org/translate/), we found a hypothetical ORF in this region that showed 99 % conservation at the amino acid level to ABUW_1486 (A1S_2218.1) (Fig. 4c). Thus, although not annotated in the ATCC 17978 genome file, we included A1S_2218.1 in our study.

**Fig. 5.**
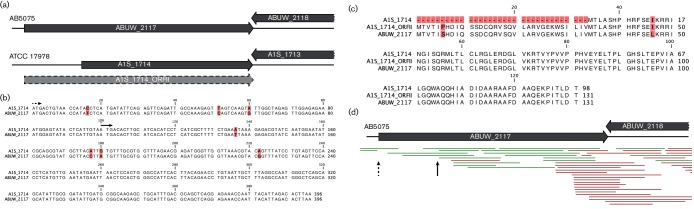
Comparison of two homologous loci reveals translational start site disagreement between strains. (a) Genomic context of the AB5075 regulator ABUW_2117 and its homologous region in ATCC 17978. Dark grey arrows highlight the annotations currently present in the genome. The annotation shown as a light grey arrow with a dashed outline marks a potentially longer ORF (A1S_1714_ORFII) in ATCC 17978. (b) Alignment of the nucleotide sequence of ABUW_2117 and the corresponding region in ATCC 17978. Pink boxes denote differences within both sequences. A black arrow with a solid line highlights the translational start site of the annotated gene, while a black arrow with a dashed line marks the potential start site of A1S_1714_ORFII. (c) Alignment of proteins encoded by ABUW_2117 and A1S_1714. The longer protein putatively encoded by A1S_1714_ORFII is shown to have only two amino acids substitutions when compared to ABUW_2117 (pink boxes). (d) Transcriptional analysis of the ABUW_2117 locus. An arrow with a solid or a dashed line, respectively, highlights the start site of the annotated and hypothetical homologues in ATCC 17978 (as shown in b). This RNA sequencing data was previously published by our group [32].

ABUW_2117 in AB5075 is a HxlR-type regulator and a homologue of A1S_1714 in ATCC 17978 (blastp = 3.7×10^−56^). Surprisingly, a corresponding nucleotide blast for these two loci returned a *E* value of 0. Upon further investigation, we found that the A1S_1714 gene is annotated with a different start codon to ABUW_2117, which results in a truncated protein (Fig. 5a, b). Interestingly, the ABUW_2117 start codon is also present in the A1S_1714 gene, and would produce a longer protein similar to that of the AB5075 version (A1S_1714_ORFII). Comparing A1S_1714_ORFII and ABUW_2117 revealed a 96 % conservation of amino acid sequence between the original and alternative start codon variants of A1S_1714 (Fig. 5c). Since we cannot exclude the possibility that the shorter protein is the correct version (meaning the AB5075 annotation is wrong), we analysed AB5075 RNAsequencing data recently generated by our group (Fig. 5d) [32]. In so doing, we demonstrated that, although expressed at a low level, AB5075 mRNA transcripts extended the full length of the ORF, suggesting the upstream start codon is in fact used by the cell. As such, we chose to include A1S_1714_ORFII as a homologue of ABUW_2117 in our study. Based on the findings presented herein, the putative A1S_2218.1 and the alternative A1S_1714_ORFII were both included in our updated ATCC 17978 annotation file.

**Fig. 6.**
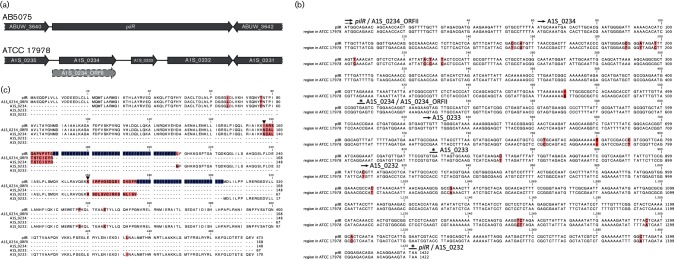
Comparison of the PilR RR in AB5075 and its homologue in ATCC 17978. (a) homologous regions in AB5075 and ATCC 17978: dark grey arrows denote current annotations in both genomes, while a light grey arrow with a dashed outline marks a potential longer ORF, A1S_0234_ORFII, in ATCC 17978. In AB5075 *pilR* is encoded as one continuous gene, while in ATCC 17978 three different genes are annotated in this region. (b) Comparison of the nucleotide sequences for both strains reveals numerous mutations, including frameshifts (red boxes) and nucleotide substitutions (pink boxes) in ATCC 170978. These changes lead to three separate genes, encoding three separate proteins, in ATCC 17978. Black arrows with solid lines highlight the translational start sites of annotated genes, an arrow with a dashed line marks the potential start site of A1S_0234_ORFII and solid lines with asterisks highlight stop codons. (c) An alignment of PilR to the three ATCC 17978 proteins shows that the frameshift mutations for A1S_0233 and A1S_0234/A1S_0234_ORFII result in alterations to the amino acid sequence at the C-terminal end of each protein (marked by black triangles), and ultimately result in premature termination of the protein. The introduction of a mutation in the nucleotide sequence (C907T) led to a new start codon, resulting in the presence of A1S_0232. Changes in the amino acid sequence are denoted by pink boxes, while areas of PilR that do not have homologous sequence in ATCC 17978 are highlighted in blue.

Finally, when performing a nucleotide blast with ABUW_3641, a putative type IV fimbriae RR, we identified a highly similar region in the genome of ATCC 17978 that harbours three separate ORFs (Fig. 6a). The nucleotide alignment of this region showed two frameshift mutations, resulting in the introduction of two stop codons, producing three distinct genes encoding different proteins. These three separate proteins all possess unique domains: A1S_0234 specifies a RR receiver domain, A1S_0233 harbours a σ^54^ interaction domain and A1S_0232 is a TF of the Fis family bearing a HTH8 domain, which is also conserved in ABUW_3641 (Fig. 6b, c). Although one can only speculate about the biological significance of this unique arrangement, one possible explanation could be that the flexibility of this tripartite regulatory system in ATCC 17978 is better suited to its niche-specific lifestyle than a single protein such as ABUW_3641 in AB5075. Given the uncertain biological relevance of this unique arrangement, we did not record this regulator as present in ATCC 17978.

### Concluding remarks

Over the last three decades, *A. baumannii* has emerged as a pathogen of major clinical concern due to its MDR nature and abundant virulence traits, enabling it to cause a wide variety of infections. Despite this, the regulatory networks present in this organism remain poorly understood, particularly with respect to pathogenesis. We have previously documented the global identification and curation of regulatory RNAs in *A. baumannii* [32]. As such, a primary goal of this study was to comprehensively explore proteinaceous regulatory factors in the representative, and highly virulent, AB5075 strain. As part of this, we combined our bioinformatic analyses and literature searches with comparison to a recent TnSeq study of AB5075 using a *G. mellonella* infection model [12]. This analysis allowed us to identify regulatory elements common to all *A. baumannii* strains, which we suggest are likely important for key biological processes, whilst at the same time uncovering those that are more narrowly possessed, potentially indicating specialized and niche-specific functions. It also allowed us to correlate conservation with a known role in virulence; thus, shedding light on the molecular basis of hypervirulence in certain clones and isolates. We further identified a number of uncharacterized regulatory elements that are not strongly conserved, including members of the Fur and LysR families, which are only present in clinical isolates of *A. baumannii*, again potentially explaining the hypervirulent phenotypes of certain strains. Collectively, this data provides novel candidates for further investigation, particularly as putative anti-virulence-based drug targets. Furthermore, our extensive genomic examination at the nucleotide level, in combination with traditional protein blasts, allowed us to detect the presence of frameshift mutations and base substitutions in several TFs. This finding suggests functional divergence of certain proteins, by which genes with novel roles may have arisen and become increasingly suited to the lifestyle of contemporary clinical isolates. In summary, this study provides a significant resource to those working in the area of *A. baumannii* regulation and pathogenesis, delivering a comprehensive overview of the proteinaceous regulome. We suggest that it provides a major foundation for the continued understanding of pathogenic mechanisms within this important human pathogen.

## Data bibliography

1. Casella LG, Weiss A, Pérez-Rueda E, Ibarra JA, Shaw LN. https://figshare.com/s/690a28e453bbe85eb683.

## Supplementary Data

85 Supplementary File 1

## Supplementary Data

52 Supplementary File 2
